# Corrosion Behavior of Nitrided Layer of Ti6Al4V Titanium Alloy by Hollow Cathodic Plasma Source Nitriding

**DOI:** 10.3390/ma16082961

**Published:** 2023-04-07

**Authors:** Lei Zhang, Minghao Shao, Zhehao Zhang, Xuening Yi, Jiwen Yan, Zelong Zhou, Dazhen Fang, Yongyong He, Yang Li

**Affiliations:** 1 School of Electromechanical Automobile Engineering, Yantai University, Yantai 264005, China; 2 State Key Laboratory of Tribology, Tsinghua University, Beijing 100084, China; 3 Department of Nuclear Equipment, Yantai University, Yantai 264005, China

**Keywords:** titanium alloys, plasma nitriding, TEM, HRTEM, electrochemical impedance spectroscopy

## Abstract

Ti6Al4V titanium alloys, with high specific strength and good biological compatibility with the human body, are ideal materials for medical surgical implants. However, Ti6Al4V titanium alloys are prone to corrosion in the human environment, which affects the service life of implants and harms human health. In this work, hollow cathode plasm source nitriding (HCPSN) was used to generate nitrided layers on the surfaces of Ti6Al4V titanium alloys to improve their corrosion resistance. Ti6Al4V titanium alloys were nitrided in NH_3_ at 510 °C for 0, 1, 2, and 4 h. The microstructure and phase composition of the Ti-N nitriding layer was characterized by high-resolution transmission electron microscopy, atomic force microscopy, scanning electron microscopy, X-ray diffraction, and X-ray photoelectron spectroscopy. This modified layer was identified to be composed of TiN, Ti_2_N, and α-Ti (N) phase. To study the corrosion properties of different phases, the nitriding 4 h samples were mechanically ground and polished to obtain the various surfaces of Ti_2_N and α-Ti (N) phases. The potentiodynamic polarization and electrochemical impedance measurements were conducted in Hank’s solution to characterize the corrosion resistance of Ti-N nitriding layers in the human environment. The relationship between corrosion resistance and the microstructure of the Ti-N nitriding layer was discussed. The new Ti-N nitriding layer that can improve corrosion resistance provides a broader prospect for applying Ti6Al4V titanium alloy in the medical field.

## 1. Introduction

For various medical applications, the ideal biomaterial is required to have properties such as excellent corrosion resistance in the body fluid medium, good biocompatibility, high strength, high ductility, low modulus, and no adverse tissue reactions [1,2,3]. Titanium alloys are used for a wide range of applications in medical implant teeth, artificial hip joints, shoulder joints, knee joints, bone fixing nuts, and screws [4,5]. Although titanium alloys have good biocompatibility, the complexity of the human environment makes titanium alloy human implants vulnerable to corrosion, which seriously affects the service life of implants. Several studies have shown that titanium alloy implants, such as artificial hip joints and knee joints, will age after 10–15 years of use [6]. At the same time, the release of Al, V, and other metal ions in titanium alloy implants is harmful to human health under the combined effect of wear and corrosion [7,8].

Different surface treatment techniques of titanium alloys can effectively provide these surfaces with more desired properties and functionalities for exceptional applications [9,10,11,12,13,14,15,16]. Electrochemical anodic oxidation technology has the advantages of rapid preparation, diversified surface morphology, and low cost. Martinez et al. [17] show that dense oxide films were developed on the surface of Ti6Al4V alloy by constant current anodizing. This film contains amorphous oxides which greatly improve the corrosion resistance of the alloy. However, the low energy utilization and high energy consumption of anodic oxidation result in high treatment costs. Laser cladding technology can form a high hard ceramic phase on the surface of the alloy and significantly improve the wear resistance of titanium alloys [18]. Zhang et al. [19] used laser remelting technology to generate hydroxyapatite (HA) coating on the Ti6Al4V surface and studied its friction, corrosion, and biocompatibility. It was found that the coating had excellent corrosion resistance and wear resistance and could promote the proliferation of bone cells. However, its equipment conditions are demanding, and the treatable area is limited, making mass production difficult. The protection of the substrate is insufficient, and cracks are easily generated. Large stress concentration exists between the modified layer and the substrate. Gordon et al. [20] modified the super elastic nickel-free titanium-based biomedical alloy by nitrogen ion implantation, reducing the material surface’s friction coefficient, and improving the material’s corrosion resistance, hardness, and biocompatibility. The ion implantation technique has no coating film base bonding problems. The injection process does not require an elevated substrate temperature, and the workpiece is not deformed. The disadvantage is that it is easy to cause lattice damage, and the ion injection layer is shallow— only 0.1~1.0 μm. During Plasma Vapor Deposition (PVD) treatment, single or multiple source materials (including oxides, carbides and nitrides) are evaporated or sputtered in a high vacuum [21,22]. Li et al. [23] prepared TiN and Ti/TiN multilayer coatings on the Ti6Al4V alloy using the PVD technique. These coatings exhibited high hardness in simulated body fluids while showing excellent corrosion resistance and relatively low friction coefficient. However, there are some disadvantages in PVD coating, such as poor adhesion with the coating and spontaneous falling under high stress.

Plasma nitriding technology has high activity, a fast penetration rate, and a deeper penetration layer that is easier to obtain [24,25,26]. In the process of plasma nitriding, ionized nitrogen bombards the surfaces of components with high cathode potential under the action of the electric field. Nitrogen ions sputter and heat the surfaces of the parts to be treated and diffuse into the parts. From the surface of the components to the substrate, the concentration of nitrogen ions gradually decreases, forming a compound layer from TiN to Ti_2_N in turn [12,27,28]. The existence of a compound layer enables titanium nitride alloys to obtain high hardness, wear resistance, and good corrosion resistance. Y.V. Borisyuk et al. [29] performed plasma nitriding treatment of the Ti5Al4V2Mo alloy at different temperatures, and the hardness of the treated samples increased by 1.7 times compared to the untreated surface. A. Sowińska et al. [24] concluded that these TiN + Ti_2_N + a-Ti(N)diffusion surface layers on the Ti-6Al-4V alloy by using plasma nitriding are a promising bio-material. Such layers of homogenous structure and thickness, and low surface free energy can be produced even on elements with complicated shapes, which is characteristic for cardiovascular implants. N. Rajendran et al. [30] reported that the nitrided b-21S titanium alloy exhibited a high-percentage of cell viability demonstrating their increased biocompatibility.

However, traditional plasma nitriding technology has unavoidable defects, such as edge effect and surface arc [31,32]. One way to avoid this problem is active screen plasma nitriding (APSN) in recent years [33,34,35]. In this method, a high negative potential is applied to a cage that generates an inner cavity filled with nitrogen plasma. All sides of the parts in the cage are kept at a floating potential, and the coating produced by exposure to hot plasma is very popular. At the same time, the processed parts are isolated from DC high voltage. However, the inflow of high-energy nitrogen ions will be offset, and the modified layer formed by ASPN will become thinner. It can be considered that the ASPN process still belongs to a linear abnormal glow discharge. If the nitriding treatment adopts hollow cathode discharge, the ionization rate and plasma density generated will be greater than the linear abnormal glow discharge [36,37]. Our previous work concluded that the HCPSN nitrided sample had a higher corrosion resistance in comparison with the bare AISI 4140 steel and the CPN samples [38]. HCPSN treatment is widely used for its fast-processing speed, low energy consumption, low part deformation and no pollution. In other studies, HCPSN was used for nitriding the Ti6Al4V alloy. However, the structure of different phases on the surface of the sample after nitriding has not been investigated further [39].

In this study, HCPSN treatment was used to modify the surface of the Ti6Al4V titanium alloy, and the microstructure of the Ti-N nitriding layer was characterized by various methods. Hank’s solution was used to simulate the human environment, and the potentiodynamic polarization and electrochemical impedance tests were conducted to characterize its corrosion resistance. The relationship between the microstructure of the Ti-N nitriding layer and the corrosion resistance was discussed. The results show that the corrosion resistance of the Ti6Al4V titanium alloy can be significantly improved by hollow cathode plasma source nitriding.

## 2. Materials and Methods

Ti6Al4V alloy samples are supplied in the form of 20 mm diameter round bars, wire cut into samples of 8 mm thickness. The chemical composition of the samples is given in Table 1. Their surfaces were ground with sandpapers and mechanically polished with the 50 nm SiO_2_ polishing solution. Then they were cleaned separately with acetone and ethanol using an ultrasonic cleaner.

**Figure 1:**
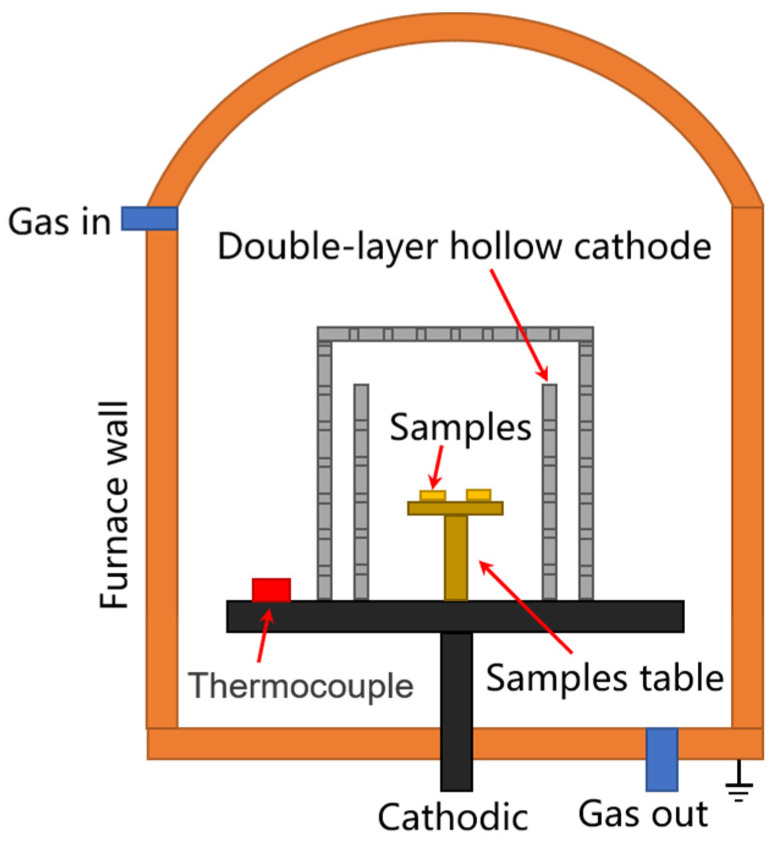
Schematic diagrams of hollow cathodic plasma source nitriding furnace device.

The hollow cathode device consists of a double-layered hollow cathode cylinder with a top cover, as shown in Figure 1. The material of the hollow cathode barrel is Ti6Al4V, with the regular arrangement of small holes of 5 mm diameter. The Ti6Al4V samples were placed in the hollow cathodic device and connected to the cathode in the nitriding furnace (LDMC-20F, WHRCLS, Wuhan, China). The plasma nitriding process was started by pumping the air pressure inside the furnace to less than 10 Pa. After that, NH_3_ was gradually introduced into the furnace until a pressure of 300 Pa was reached. The nitriding furnace was maintained at a pulse frequency of 1000 Hz, a duty cycle of 70% and a voltage of 700 V which activated the glow discharge process. The process was conducted at a temperature of 510 °C. Finally, the samples were cooled with the furnace in vacuum. The preparation process of different samples is shown in Table 2.

An optical microscope (OM, ZEISS Axio Observer 3 materials, Oberkochen, Germany) was used to analyze the microstructure of samples. The phase constitutions of the samples were determined using X-ray diffraction (XRD, Bruker D8 ADVANCE, Billerica, MA, USA) with a Cu-Kα radiation source. The scanning angle ranged from 20° to 90 with a rate of 4°/min, and the accelerating voltage was 40 kV. The surface phases of the untreated and corroded samples were analyzed with X-ray photoelectron spectroscopy (XPS, PHI Quantera II, Japan). The XPS measurements were based on a classic X-ray optical scheme. Electrostatic focusing and magnetic screening were used to achieve an energy resolution of ΔE ≤ 0.5 eV for the Al Kα radiation (1487 eV). The XPS spectra were recorded with the 100 μm X-ray spot, and the X-ray power supplied to the sample was 23.6 W. All XPS measurements were performed with argon ion cleaning. Data were analyzed by Casa XPS software. The C-C binding energy was calibrated for the peak spectrum at 284.8 eV. The split peak fitting of the XPS peaks was performed based on a Shirley-type baseline. The microstructure of the near-surface region of the samples was observed using a transmission electron microscope (TEM, JEOL JEM-2100, Japan). The imaging was performed in a bright field (TEM/BF). The phase analysis was performed with the help of DM3 software based on selected area electron diffraction (SAED) patterns and high-resolution transmission electron microscopy (HRTEM). Energy dispersive X-ray spectroscopy (EDS) was used to determine the distribution of the surfaces’ chemistry. The samples for TEM were cut out using the focused ion beam (FIB, FEI Quanta 200 FEG, Hillsboro, OR, USA) technique. The Vickers hardness test was performed on the surfaces of samples using a Vickers hardness tester (Struers Duramin-A300) with the test load of 50 g and the holding time of 15 s. The surfaces were measured multiple times at each test point.

A series of electrochemical tests, including dynamic potential polarization and electrochemical impedance, were performed with the assistance of an electrochemical workstation (VersaSTAT 3F, Berwyn, PA, USA) to assess the corrosion behavior of the nitriding sample in the cathode environment of Hank’s solution. The composition (g/L) of Hanks solution is NaCl 8, KCl 0.4, CaCl_2_ 0.14, MgSO_4_·7H_2_O 0.2, Na_2_HPO_4_·H_2_O 0.09, KH_2_PO_4_ 0.06, NaHCO_3_ 0.35, and C_6_H_12_O_6_ 1.0. A three-electrode system was used for corrosion testing. Among them, samples, saturated calomel electrodes (SCE, saturated KCl), and platinum electrodes were used as working electrodes, reference electrodes, and counter electrodes, respectively. Prior to all experiments, the untreated Ti6Al4V and nitriding samples were immersed in Hank’s solution at open circuit potential (OCP) for 1 h to achieve electrochemical stability. The potential polarization was tested from −1.0 V_SCE_ to 1.0 V_SCE_ with a scan rate of 0.5 mV/s. EIS measurements were performed at OCP with a frequency of 10^5^ to 10^−2^ Hz and an amplitude of 10 mV.

## 3. Results

**Figure 2:**
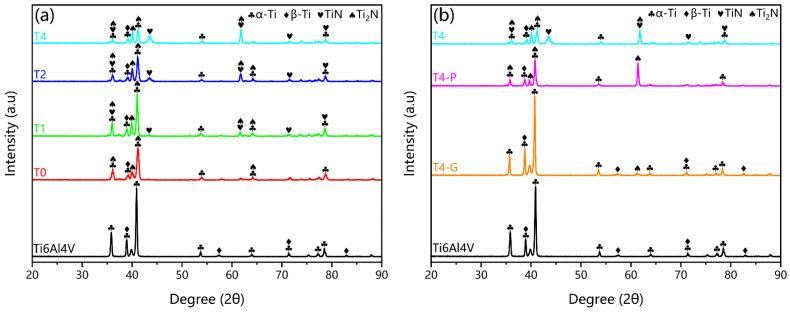
(**a**) The XRD patterns of the untreated Ti6Al4V, T0, T1, T2, and T4 samples; (**b**) The XRD patterns of the untreated Ti6Al4V, T4-G, T4-P, and T4 samples.

The XRD patterns of untreated Ti6Al4V and different nitriding time samples are shown in Figure 2a. The three main peaks were at 35.1°, 38.4°, and 40.2°, corresponding to the (10$\bar{1}$0), (0002), and (10$\bar{1}$1) phases of α-Ti (JCPDS 44-1294), respectively [40]. The α-Ti has a hexagonal close-packed (HCP) structure. The β-Ti was mainly in the (110) phase at 38.5° (JCPDS 44-1288), with a body-centered cubic (BCC) structure. All α-Ti and β-Ti peaks decreased with increasing nitriding time. The Ti_2_N phase has been detected in the sample with a holding time of 0 h for T0 sample. According to the theory of nitrogen diffusion in titanium, nitrogen first entered the matrix as an interstitial solid solution. When the concentration reached a certain value, Ti_2_N was generated at the surface. This process can be accelerated by using the hollow cathode plasma source. The (200) and (220) phases of TiN appeared at 42.6° and 61.8° for the T1 samples (JCPDS 38-1420), and the peaks increased with increasing nitriding time.

Figure 2b shows the XRD patterns of the Ti-N nitriding layer with different surface phases obtained by mechanical grinding and polishing methods. The T4-G sample has an overall shift of 0.5° to a small angle compared to the peak of the untreated Ti6Al4V sample, which is a common shift in solid solution XRD of nitrogen. The T4-P sample shows a significant increase in the (002) phase at 61° compared to the T0 sample in Figure 2a. This indicated that the newly generated Ti_2_N phase on the surface of the T0 sample was different from the Ti_2_N layer at the bottom of the TiN layer on the surface of the T4 sample by HCPSN treatment.

**Figure 3:**
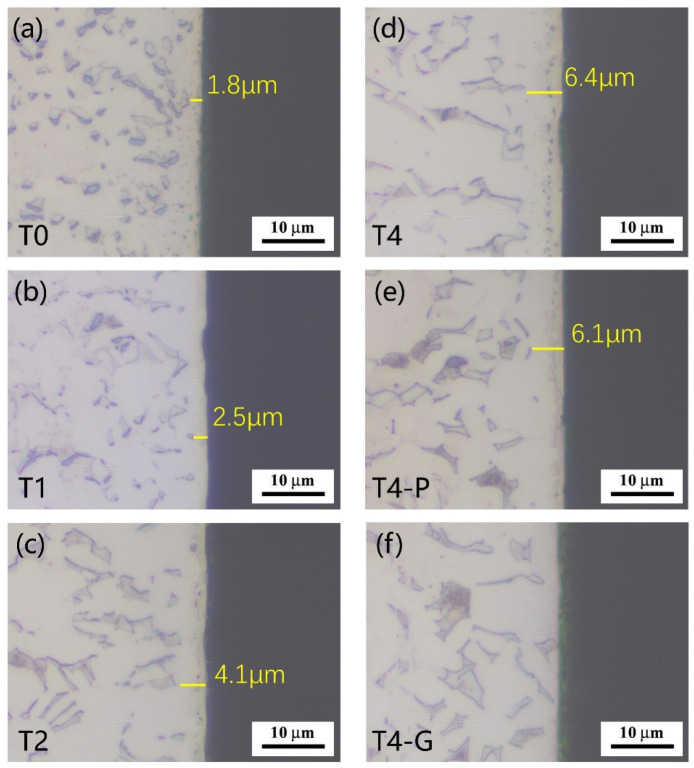
The cross-sectional optical micrographs of different samples.

The cross-sectional optical micrograph of the sample is shown in Figure 3. The thickness of the compound layer increased significantly with the increase in nitriding time of the hollow cathode plasma source. The compound layer thicknesses of T0, T1, T2, and T4 samples were about 1.8, 2.5, 4.1, and 6.4 μm, respectively. According to the previous study [41], the thickness of the TiN layer is about 300~500 nm. Therefore, the corresponding thickness was removed by mechanical polishing on the basis of T4 samples, as shown in Figure 3e. The XRD pattern shows that the TiN phase is no longer present on the surface of this sample and is replaced by Ti_2_N. Similarly, the T4-G samples were obtained by removing 6~7 μm on the surface of the T4 samples, as shown in Figure 3f.

**Figure 4:**
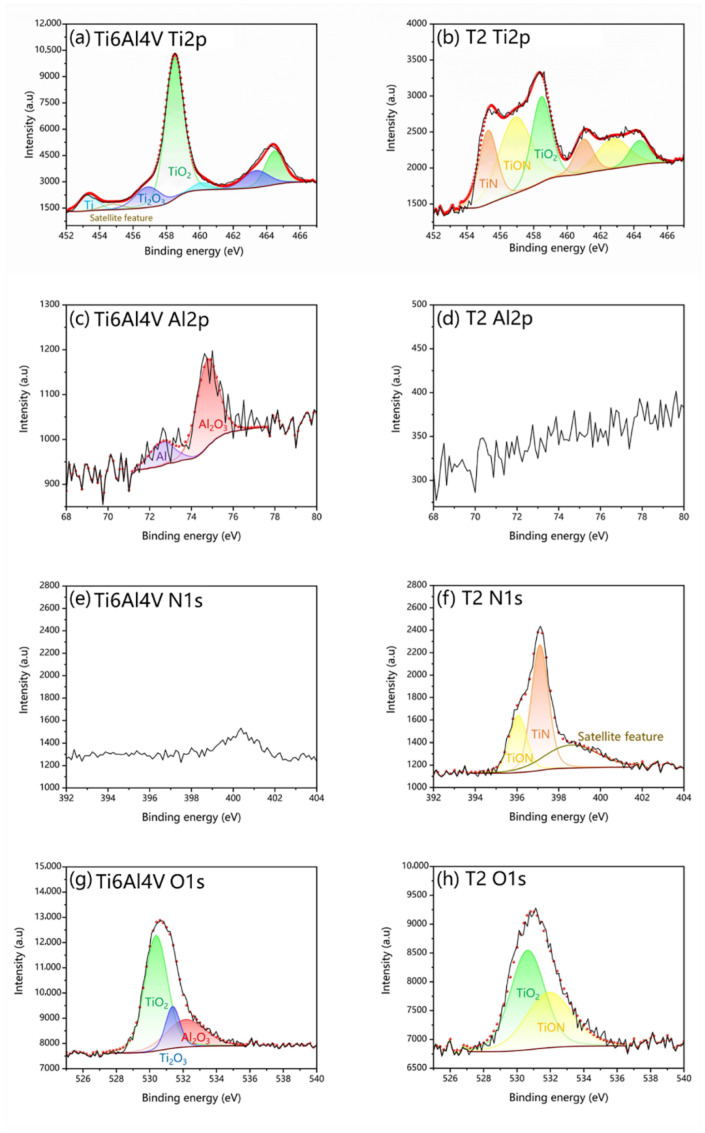
The XPS images of untreated Ti6Al4V: (**a**) Ti 2p, (**c**) Al 2p, (**e**) N 1s, (**g**) O 1s; and T2 samples: (**b**) Ti 2p, (**d**) Al 2p, (**f**) N 1s, (**h**) O 1s.

The XPS images of the untreated and T2 samples are shown in Figure 4. The spectra of Ti2p, Al2p, N1s, and O1s were separated into peaks, respectively. The Ti2p_3/2_ of the untreated Ti6Al4V sample had three peaks at binding energies of 453.2 eV, 456.9 eV, and 458.5 eV for Ti, Ti_2_O_3,_ and TiO_2,_ as shown in Figure 4a. The N1s peak spectrum has no distinct peaks, as in Figure 4e. Figure 4g shows that the corresponding O1s peak spectrum has three characteristic peaks for TiO2, Ti_2_O_3,_ and Al_2_O_3_ [42]. The Ti2p_3/2_ in the surface layer of the T2 nitride sample was fitted to three representative peaks, respectively, and corresponding groups were TiN, TiON, and TiO_2_ [43]. The presence of Al and Al_2_O_3_ was confirmed in Figure 4c [44]. Figure 4d indicated that there was no Al elemental present on the surface of the T2 sample and that Al migrated to the core of the sample, which needs to be discussed separately. The N1s peak spectrum has two correlation peaks at 396.1 eV and 397.3 eV, corresponding to TiON and TiN, as shown in Figure 4f [45]. TiO_2_ and TiON were detected in the O1s peak spectrum on the surface of the nitriding samples, as shown in Figure 4h.

**Figure 5:**
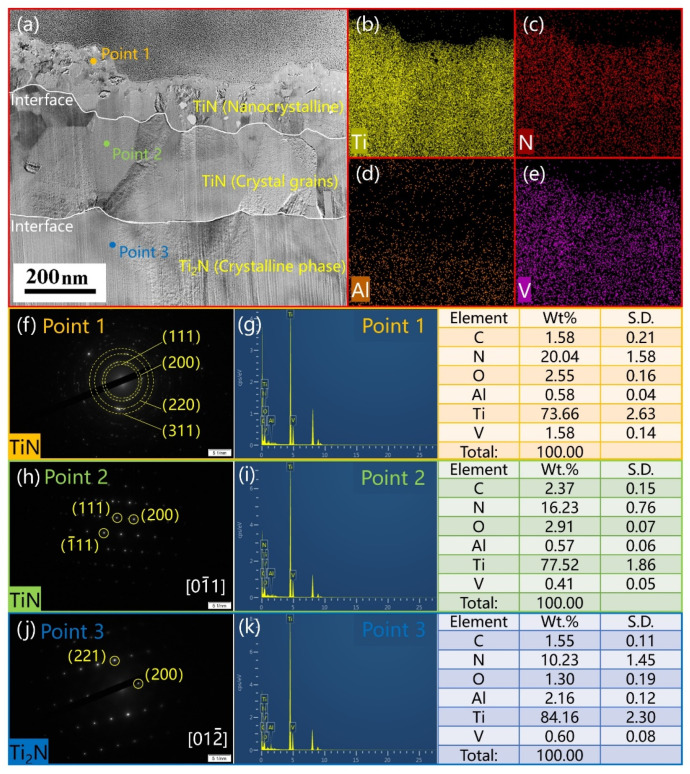
(**a**) Cross-sectional TEM image of sample 510-2. The most superficial layer of the sample is nanocrystalline TiN, and the subsurface layer is the crystalline TiN layer. The lower layer of the TiN layer is the Ti_2_N layer. The interface between the layers is obvious. EDS surface scan was performed for the whole area as shown in (**b**) Ti, (**c**) N, (**d**) Al, (**e**) V. The selected area electron diffraction (**f**–**j**), and EDS point scan (**g**–**k**) were performed for Points 1, 2, and 3. The tables show the results of the point scan. The data were averaged over multiple measurements, and the standard deviation (S.D.) was calculated.

The cross-sectional TEM image of the T2 sample is shown in Figure 5a. The EDS surface scan of the whole area is shown in Figure 5b–e. The Ti element content was high in the whole sample. The N element content decreased gradually from the outer layer to the inner part, while the opposite was true for the Al element. This was due to the migration of Al elements to the inner part of the sample. The HCPSN nitriding of the Ti6Al4V alloy pushed aluminum away from the near-surface and enriched the Ti_3_Al, forming the Ti_3_Al intermetallic phase [46]. The SEAD analysis was performed on Points 1, 2, and 3 in Figure 5a. The results are shown in Figure 5f–j. The typical nanocrystal diffraction ring is shown at Point 1. The (111), (200), (220), and (311) phases of TiN are shown from inside the center of the circle outward, respectively. The SEAD diagram at Point 2 is shown in Figure 5h. The composition of Point 2 was the (111), (−111), (200) phase of crystalline TiN on the [0, −1, 1] crystal band axis. Point 2 had the typical diffraction morphology of twin crystals. The (211), (200) phase of Ti_2_N is shown in Figure 5j at Point 3. Activation of the nitridation reaction by hollow cathode assistance leads to more efficient nitridation [47]. The most superficial layer of the sample was nanocrystalline TiN, and the subsurface layer was the crystalline TiN layer. The lower layer of the TiN layer was the Ti_2_N layer. The surface microstructure of hollow cathodic nitriding samples at 500 °C is similar to the result of conventional plasma nitriding at 850 and 900 °C studied by Czyrska-Filemonowicz et al. [48]. Furthermore, short-time nitriding assisted by hollow cathodes avoids surface defects of plasma nitriding (e.g., nano-whiskers of Ti and Al oxides [49]). The interface between the layers was obvious. Extended exposure to high temperatures during longer nitriding times increases grain growth [50]. The results of the point scan in Figure 5g–k confirmed this idea. The Wt. % of each component is shown in the table. These data were the average of multiple measurements. The standard deviation (S.D.) was also calculated.

**Figure 6:**
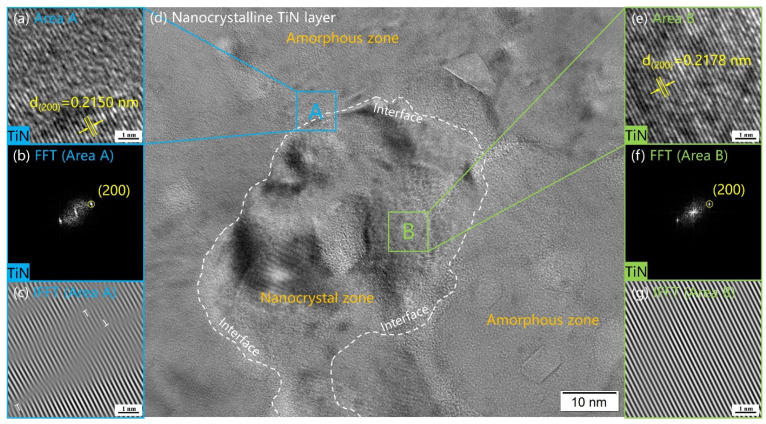
The HRTEM image (**d**) of surface layer nanocrystalline TiN. The most superficial layer was a nanocrystalline TiN layer. The TiN nanocrystals were surrounded by amorphous zone. The crystals were irregularly shaped and had a diameter of about 50~100 nm. The FFT (**b**) and IFFT (**c**) images of Area A (**a**). The FFT (**f**) and IFFT (**g**) images of Area B (**e**).

The HRTEM images of the surface layer of nanocrystalline TiN are shown in Figure 6d. The most surface layer was the nanocrystalline layer of TiN (200). The amorphous zone surrounded the TiN nanocrystals. The shape of the crystals was irregular, and the diameter was about 50~100 nm. Hollow cathodes have high activity and a high density of plasma inside, and they are more likely to adsorb on the sample surface. The large number of incoming high-energy N^+^ ions from the hollow cathode causes many surface defects, providing more nucleation sites with different orientations than in the case of conventional nitriding methods [51]. The dislocation density increases, thus promoting the refinement of high free energy grain size on the top surface and accelerating the diffusion of nitrogen [52]. The accelerated nitrogen diffusion kinetics leads to the formation of nanocrystalline TiN layers on the top surface and increases the nitrogen diffusion depth. This is attributed to the higher density of crystal defects in the nonequilibrium nanocrystalline TiN layer, which increases the surface energy storage of reactive nitrogen and its chemical reactivity, providing additional preferential nucleation sites for TiN [53].

**Figure 7:**
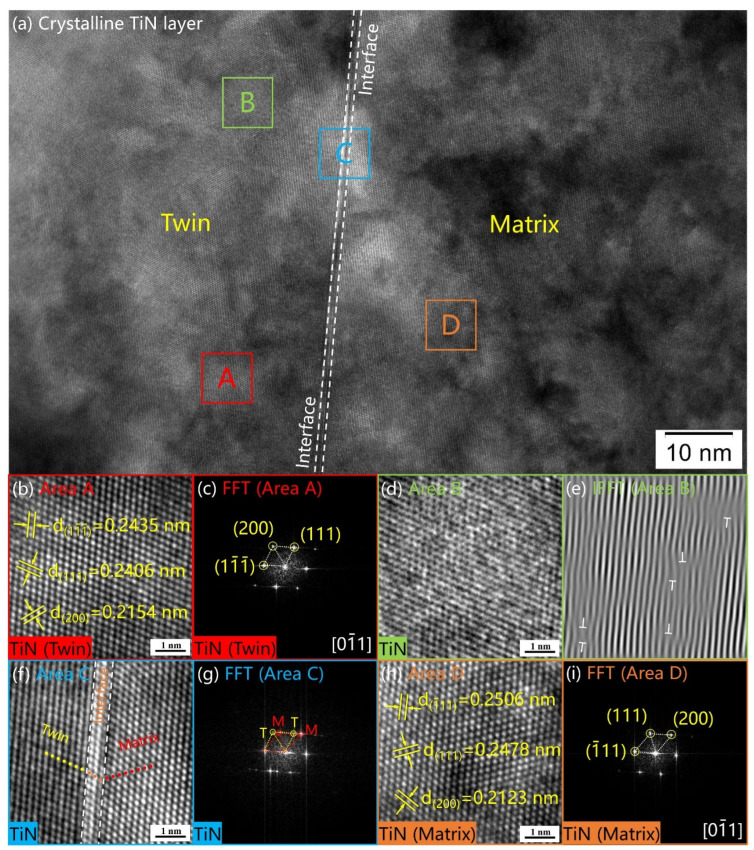
(**a**) The HRTEM image of crystalline TiN layer. With the interface in figure (**a**) as the boundary, the left side is the twin crystal, and the right side is the matrix; (**b**) The image of twin crystal in area A; (**c**) The FFT image of area A; (**d**) The image of area B; (**e**) The IFFT image of area B. There are a large number of dislocations distributed in area B; (**f**) The image of the interface in area C; (**g**) The FFT image of area C; (**h**) The image of the matrix in area D; (**i**) The FFT image of area D.

The HRTEM images of the subsurface layer are shown in Figure 7a. The enlarged HRTEM image of Area A is shown in Figure 7b, and the FFT image of Area A is shown in Figure 7c. Taking the interface in Figure 7a as the demarcation, the TiN matrix was on the right, and the twin crystal of TiN was on the left. In the [0–11] crystal zone, three crystal plane groups were detected with lattice spacings of 2.435, 2.406, and 2.154 Å. They are close to the (111) and (200) crystal planes of TiN (2.45Å and 2.12 Å, JCPDS 38-1420). There is a large amount of dislocation buildup inside the twins, as shown in Figure 7e. Dislocations affect the mechanical properties and corrosion resistance of nitrided samples. At the interface (Area C), the crystal plane undergoes a small angle shift, as shown in Figure 7f. Figure 7g showed a typical twin crystal diffraction dot pattern. The crystalline planes with lattice spacings of 2.506 Å, 2.478 Å, and 2.123 Å were observed in the TiN matrix, corresponding to (−111), (111), and (200) crystallographic planes (2.45Å and 2.12 Å, JCPDS 38-1420). The matrix and twin are symmetrical about the (−111) crystal plane. The superficial nanocrystalline TiN layer regulates the subsurface crystalline TiN by activating twinning and dislocation mechanisms. Grain boundaries, dislocations, layer faults, and twins act as diffusion shortcuts by providing convenient diffusion channels for interstitial nitrogen atoms [53].

**Figure 8:**
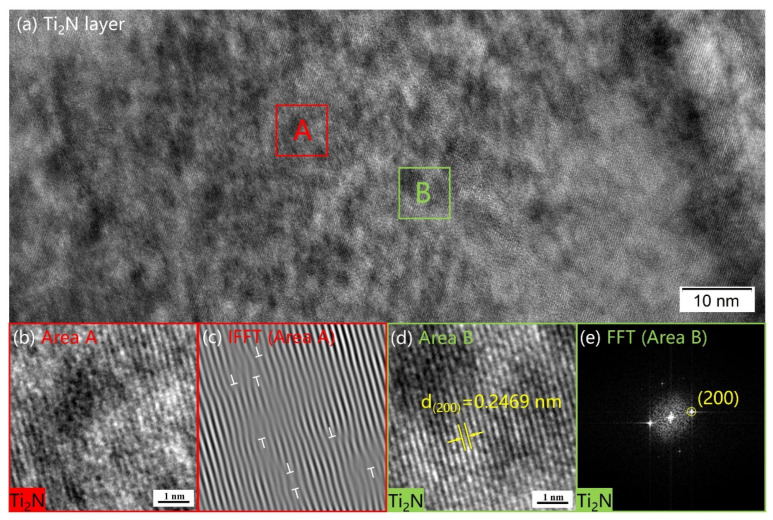
(**a**) The HRTEM image of the Ti_2_N layer; (**b**) The image of area A; (**c**) The IFFT image of area A. Large dislocation distribution within Ti_2_N layers; (**d**) The image of area B; (**e**) The FFT image of area B.

The microstructure of the Ti_2_N layer can be observed by HRTEM images, as in Figure 8. There are a large number of dislocations inside the Ti_2_N layer. The lattice spacing was detected to be 2.469 Å, which is close to the lattice spacing of the (200) crystal plane of Ti_2_N (2.47 Å, JCPDS 17-0386). The bottom boundary of TiN fluctuates from below along the top of the larger grains. At higher temperatures assisted by hollow cathodes, they also show that the growth of TiN generated in the nitriding treatment proceeds both toward the core of the sample, controlled by inward diffusion from the bottom of the TiN layer to form Ti_2_N, and to a lesser extent toward the surface, controlled by outward diffusion of titanium [51]. On the other hand, the stresses caused by the difference in thermal contraction between TiN and α-Ti(N) after nitride cooling lead to late transformation to strain-induced Ti_2_N [35].

**Figure 9:**
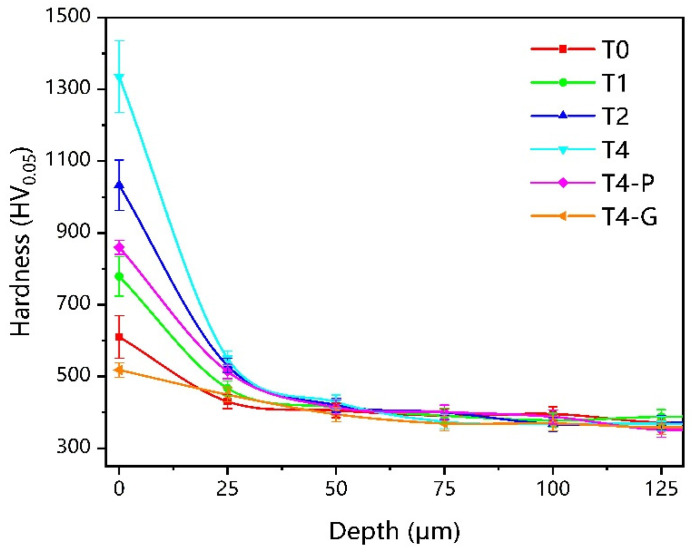
The cross-sectional hardness profile of different samples.

Figure 9 shows the cross-sectional hardness profile of nitriding, mechanically ground, and polished samples after nitriding. After nitriding, the surface hardness of the sample was increased. The surface hardness of T4 samples averaged 1340 HV_0_._05_, while T2 samples also had a surface hardness of 1032 HV_0_._05_. As the nitriding time increases, the surface hardness increases, and, at the same time, the error value of the hardness value increases. The error range of T4 samples was approximately 190 HV_0_._05_, while the error range of T0 samples was 48 HV_0_._05_. For the T4-G sample, the error range is only 26 HV_0_._05_. The surface phase of the T4-P sample is Ti_2_N, with a hardness of 865 HV_0_._05_, which is higher than the surface hardness of the T1 sample. The diffusion layer thickness was around 25 μm for the T0 sample and between 50μm and 60μm for the rest of the samples. The grain size reduction due to hollow cathodic nitriding results in a fine grain strengthening effect [54]. The gradient structure of the nitriding layer nano-TiN, crystalline TiN, Ti_2_N, and α-Ti(N) is the main reason for the increase in the hardness of the samples. The nano-structure of the TiN layer and the generation and accumulation of dislocations within the grains are effective ways to increase the surface strength of the samples [55]. The distribution of twins within the crystalline TiN results in a higher hardness of the TiN layer than the Ti_2_N layer. The surface hardness is also related to the thickness of each layer, which eventually leads to the following arrangement of surface hardness from largest to smallest: T4 > T2 > T4-P > T1 > T0 > T4-G.

**Figure 10:**
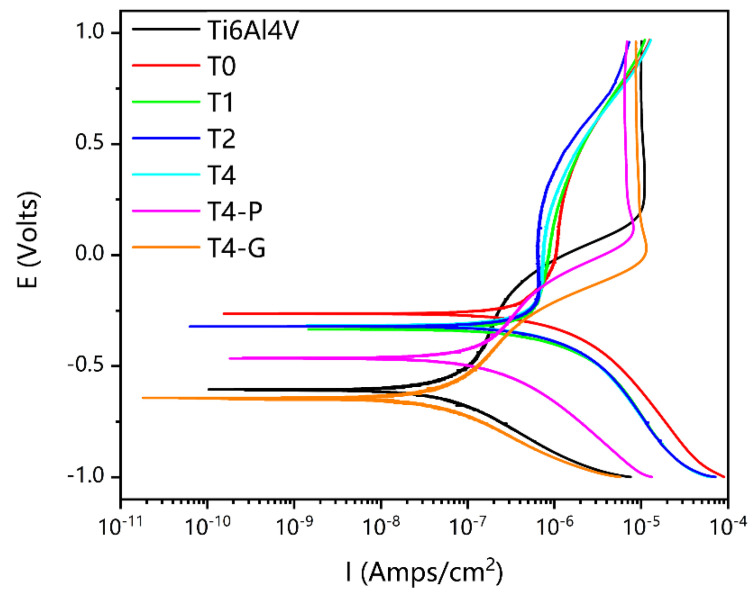
The images of the polarization curves in Hank’s solution.

The untreated Ti6Al4V and nitriding samples were measured using the dynamic potential polarization method in Hank’s solution. The results of the measured polarization curves are shown in Figure 10. The corrosion currents were obtained using the Tafel extrapolation method [56]. The corrosion rate is calculated by Equation (1) [57]. The a is the molecular weight of the electrode material; i_corr_ is the corrosion current; n is the number of equivalent exchange; F is the Faraday’s constant; and ρ is the density of the electrode material:

(1) $$\mathrm{Corrosion}\;\mathrm{rate}\;(\mu m/\mathrm{year})\;=\;3.1536\times 10^{5}\frac{ai_{\mathrm{corr}}}{\mathrm{nF}\rho }$$

The I_corr_, E_corr_, and corrosion rates of different samples are shown in Table 3. The non-mechanically treated samples after nitriding had a more pronounced passivation zone at I = 10^−6^ A/cm^2^. However, the untreated samples and the T4-P and T4-G samples had the passivation zone around I = 10^−5^ A/cm^2^. The trends of the polarization curves of the T4-P and T4-G samples were similar. It is presumed that the surface of all these samples formed an oxide film of Ti. For the nitriding samples without mechanical treatment, the polarization curves were basically the same. The TiN layer on the surface, instead of the oxide layer, acted as a good barrier and alleviated the corrosion level of the substrate [58]. It can be further demonstrated that a small amount of TiN phase on the sample surface of T0 was not detected by XRD. In terms of self-corrosion currents, the untreated T2 and T4 samples have approximated I_corr_ values. However, the I_corr_ values of the mechanically treated samples were all elevated to different degrees. This is due to the loss of the corrosion inhibition mechanism of the TiN layer. In addition, the nitride of Ti and α-Ti(N) interstitial solid solution prevented the formation of the oxide layer to some extent. In nitriding samples at different times, I_corr_ and nitriding time were negatively correlated, which was directly related to the thickness of the nitriding layer. Both sufficient TiN layer and oxide layer can reduce the I_corr_ of the substrate, but when the nitride layer is too thin, or the barrier effect of the oxide layer is weakened due to the influence of N elements, the I_corr_ will be reduced. All E_corr_ values of the samples were ranked from smallest to largest as T4-G < Untreated Ti6Al4V < T4-P < T4 < T2 < T1 < T0. For E_corr_, the nitride of Ti plays a dominant role. TiN is clearly superior to Ti_2_N and TiO_2_. A thinner nitriding layer increased the corrosion rate of the substrate, as shown in Table 3 for samples T0 and T1. The corrosion rate of the T2 sample was slightly lower than that of the Ti6Al4V untreated sample. Nitriding for 4 h, the thickness of the nitriding layer increased. However, the corrosion rate decreased. It is inferred that the deterioration of the surface layer is due to the effect of roughness. In comparison with the TiN phase and Ti_2_N phase on the surface, the corrosion resistance decreased due to lower N concentration. The interstitial solid solution phase of surface α-Ti(N) generated an oxide barrier layer of Ti, so the corrosion resistance was improved to some extent.

**Figure 11:**
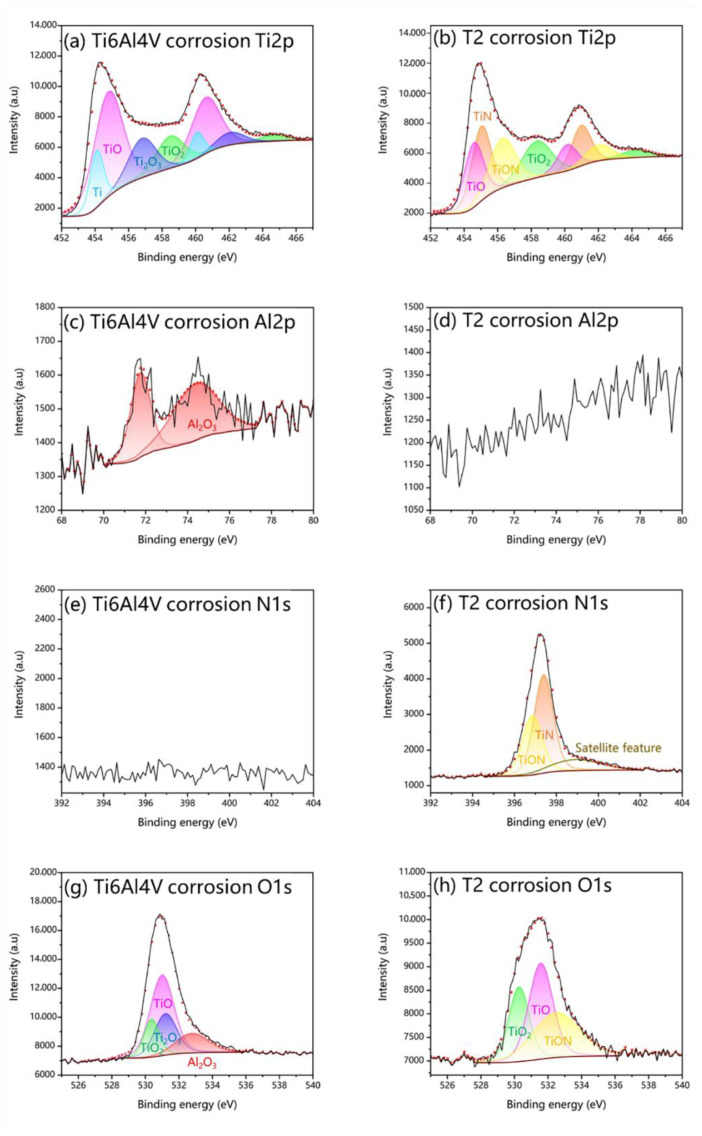
The XPS images after corrosion of untreated Ti6Al4V: (**a**) Ti 2p, (**c**) Al 2p, (**e**) N 1s, (**g**) O 1s; and T2 samples: (**b**) Ti 2p, (**d**) Al 2p, (**f**) N 1s, (**h**) O 1s.

The XPS of the untreated Ti6Al4V and T2 samples after corrosion testing confirmed a new generation of TiO, as shown in Figure 11a,b [59]. The TiO_2_ barrier on the sample surface was severely damaged, and the TiO_2_ characteristic peak area in the Ti2p peak spectrum was reduced. The dense TiN layer blocks the further occurrence of corrosion reactions. At the same time, some oxides (TiO, TiO_2_) are formed on the surface of TiN [60]. Because of the good conductivity of TiO, TiO_2,_ and TiN, galvanic corrosion will occur between the nitriding layer and the substrate [61]. When the nitriding layer is not enough to block the corrosion medium, and the substrate is exposed to corrosion, the local corrosion is accelerated to a certain extent as an anode. Therefore, the corrosion rate of T0 and T1 samples is higher than that of untreated Ti6Al4V samples.

**Figure 12:**
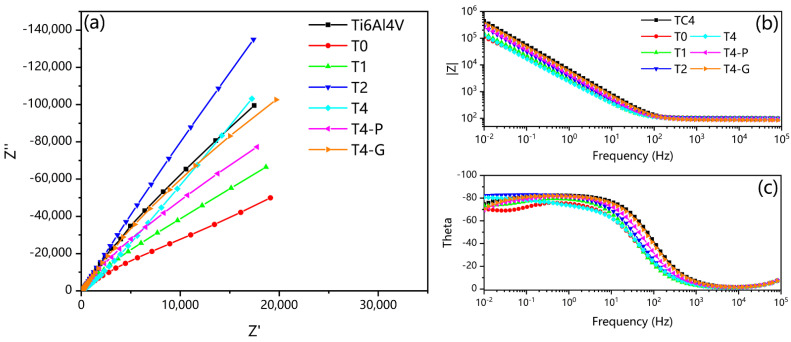
Images of EIS curves for untreated Ti6Al4V, nitriding, and mechanically treated samples: (**a**) Nyquist plots: The impedance arc radius represents the corrosion resistance of the specimen. Overall, for the nitriding samples of different times, the thicker the nitriding layer is, the better the corrosion resistance is; (**b**,**c**) Bode plots: the high-er |Z| values in the low-frequency region and wider theta peak widths in the mid-frequency region suggest better corrosion resistance.

The EIS curves of different samples are shown in Figure 12. Figure 12a shows the Nyquist plot, and the impedance arc radius represents the corrosion resistance of the specimen. The corrosion resistance of each sample was good, and the impedance values were high, so the impedance arc did not form a complete half-arc, but only a section of the half-arc. Overall, for the nitriding samples of different times, the thicker the nitriding layer is, the better the corrosion resistance is. However, when the thickness of the nitriding layer is sufficient to block the adsorption of Cl^−^ ions, the surface roughness of the sample becomes the dominant factor. The sample surface showed a high roughness level in long-time plasma nitriding due to exposing the sample surface to continuous plasma bombardment by high temperature and high voltage in the furnace [62]. The samples in the study were subjected to intense bombardment by the plasma, and it is presumed that the surface roughness of the samples showed a high deterioration with time. Due to the effect of surface roughness, the sample impedance arc radius of T4 samples is effective with that of T2 samples. The Bode plots are shown in Figure 12b,c. The higher |Z| values in the low-frequency region and wider theta peak widths in the mid-frequency region suggest better corrosion resistance [63]. For the surface phase, the effective TiN (sufficient thickness and low roughness) has the best corrosion resistance, followed by the oxides of Ti naturally occurring on the surface of untreated Ti6Al4V samples in the air. This is followed by the T4-G samples with the surface of the α-Ti(N) phase. Due to the high oxidation activity of Ti, it is presumed that a small amount of TiO_2_ will form on the surface of T4-G samples in the air to protect the substrate from corrosion. The T4-P samples with the surface of Ti_2_N phase have the worst corrosion resistance.

**Figure 13:**
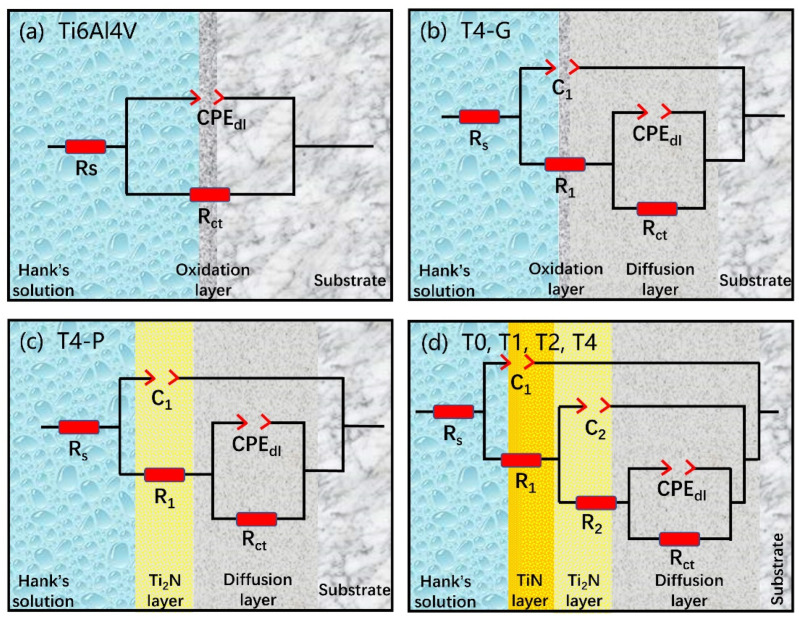
Simulated impedance circuit diagrams applied to different samples: (**a**) untreated Ti6Al4V samples. The simple impedance circuit with a time constant was used for the untreated samples to simulate the oxide layer resistance; (**b**) T4-G samples with surface mechanical grinding and polishing. A double-layer time-constant circuit was used to simulate; (**c**) T4-P samples with surface polishing; (**d**) T0, T1, T2, T4 nitriding samples. The unmechanically treated nitriding samples were fitted with a 3-time constant circuit.

Based on the EIS curve, the simulated impedance circuit diagram is shown in Figure 13. The simple impedance circuit with a time constant was used for the untreated samples to simulate the oxide layer resistance, as shown in Figure 13a [22]. The surface phase of the mechanically ground and the polished sample was inferred from the polarization and EIS curves to be α-Ti(N) with an oxide layer blocked. A double-layer time-constant circuit was used to simulate, as shown in Figure 13b [64]. Figure 13c shows the fitted circuit diagram of the mechanically polished sample with surface Ti_2_N phase. The unmechanically treated nitriding samples were fitted with a 3-time constant circuit, with R_1_ representing the resistance of the top surface layer, R_2_ representing the resistance of the subsurface layer, and R_ct_ representing the resistance of charge transfer resistance. The R_s_ represented the corrosion resistance of the seawater solution [65]. The constant phase element (CPE) was introduced to replace the ideal capacitor, as demonstrated in previous studies [41]. CPE_1_, CPE_2_, and CPE_dl_ represent the capacitance of each layer, respectively [58]. The values of the fitted circuit are shown in Table 4.

## 4. Discussion

Among the different samples, the highest R_ct_ value was obtained for the T2 sample. In general, the high R_ct_ indicates better corrosion resistance, which acts as a physical barrier to retard the penetration of ions [66]. The R_ct_ values of the nitriding samples were generally higher than those of the untreated samples. The strong and relatively deep TiN nitride layer formed during this treatment at higher temperatures can be used as a tight corrosion protection barrier [34]. The T4-P samples with the Ti_2_N surface after mechanical polishing lost the barrier of TiN on most surfaces of the nitriding layer with the lower R_ct_ value. The R_ct_ values of T4-G and untreated Ti6Al4V samples were close, and they both received protection from the surface TiO_2_ oxide layer. H. Wang et al. found that N-TiO_2_ coatings obtained by oxidative annealing after nitriding were superior to TiN and TiO_2_. This finding may be attributed to the multilayer structure of N-TiO_2_ (external oxide layer and internal diffusion zones of Ti and N), which can hinder the diffusion of reactive ions [67]. In this study, the TiO_2_ in the outer layer of α-Ti(N) plays a similar role. However, the corrosion resistance is not as good as that of the T2 sample because it forms naturally at room temperature.

In electrochemical corrosion tests, oxygen absorption corrosion occurred mainly in neutral electrolytes. The chemical reactions occurring on the surface of untreated samples are shown as follows [66]:

2TiO_2_ + 8Cl^−^ → 2TiCl_4_ + 2O_2_ + 8e^−^ (2)

Pitting corrosion occurred on the sample surface, and the TiO_2_ layer barrier protected the substrate. The pitting pits gradually expanded and increased, and the corrosion solution reacted chemically with the substrate:

Ti + O_2_ + 2H_2_O → Ti(OH)_4_ (3)

For the Ti-N nitriding layer, the reaction equation in the corrosion solution is usually [60]:

A small part of the TiN reaction generates Ti oxide:

2TiN + 4O_2_ → 2TiO_2_ + N_2_ (4)

Ti^4+^ + 2e^−^ → Ti^2+^ (5)

The reaction equation of TiN participating in corrosion is as follows:

2TiN + 8Cl^−^ → TiCl_4_ + N_2_ + 8e^−^ (6)

2TiN + 8H_2_O → 2Ti(OH)_4_ + N_2_ + 4H_2_ (7)

To conclude, a sufficient Ti-N nitriding layer acts as a barrier layer to block the intrusion of the corrosion solution. The T2 samples showed strong corrosion resistance. When the Ti-N nitriding layer is thin, the sample surface of TiN and the oxide of Ti work together. T0 and T1 samples undergo galvanic corrosion, deteriorating the corrosion resistance of the samples. The sample surface roughness also affects the corrosion resistance. The corrosion resistance is weakened by the high roughness surface caused by the T4 sample being subjected to a long and intense bombardment sputtering inside the hollow cathode. Moreover, the T4-P and T4-G samples showed different corrosion resistance, both worse than the untreated Ti6Al4V. The surface of the T4-G sample with the α-Ti(N) phase generated a small amount of TiO_2_ oxide layer to protect the substrate to some extent.

## 5. Conclusions

The microstructure, hardness, as well as electrochemical behaviors of the Ti-N nitriding layer of Ti6Al4V obtained by the HCPSN at 510 °C were investigated in detail. The main conclusions were drawn as follows:

1. XRD and TEM results show that the the Ti-N nitriding layer consists of a mixture of TiN, Ti_2_N, and α-Ti(N) phases. The compound layer consists of the nanocrystalline TiN surface top layer, the crystalline TiN sub-surface layer, the Ti_2_N interlayer, and the interstitial solid solution α-Ti(N) bottom layer. The compound layer thicknesses of the 0, 1, 2, and 4 h samples were 1.8, 2.5, 4.1, and 6.4 μm, respectively.
2. The thickness of the diffusion layer of the T0 sample was about 25 μm, and that of the remaining samples ranged from 50 μm to 60 μm. The surface hardness increased with the increase in nitriding time. The surface hardness of the T4 sample was 1340 HV_0_._05_ on average, and the surface phase of the T4-P sample was Ti_2_N with a hardness of 865 HV_0_._05_. The surface hardness of the T4-G sample was 518 HV_0_._05_.
3. The polarization results in the human body solution showed that the the Ti-N nitriding layer showed a significant passivation zone, a significant reduction in corrosion rate, and a significant improvement in pitting resistance compared to the untreated Ti6Al4V alloy. The corrosion resistance of the nitriding layer with the Ti_2_N phase on the surface deteriorated relatively. All Ecorr values of the samples were ranked from smallest to largest as T4-G < Untreated Ti6Al4V < T4-P < T4 < T2 < T1 < T0. The EIS analysis shows that the passivated film on the surface of the TiN nitride layer has higher charge transfer resistance and lower capacitance, which can effectively hinder the penetration and migration of reactive ions. Thus, the corrosion resistance is significantly improved.

## Figures and Tables

**Table 1:** Chemical composition of the Ti6Al4V in wt. %, Ti balance.

Element	Fe	O	C	N	H	V	Al
wt. %	≤0.25	≤0.18	≤0.05	≤0.05	≤0.012	3.5–4.5	5.5–6.75

**Table 2:** The preparation process of different samples.

Sample	Temperature	Heating Up Time	Holding Time	Mechanical Process
Ti6Al4V	-	-	-	grinding + polishing
T0	510 °C	45 min	0 h	-
T1	510 °C	45 min	1 h	-
T2	510 °C	45 min	2 h	-
T4	510 °C	45 min	4 h	-
T4-P	510 °C	45 min	4 h	polishing
T4-G	510 °C	45 min	4 h	grinding + polishing

**Table 3:** The I_corr_, E_corr,_ and corrosion rate of different samples.

	I_corr_ (A/cm^2^)	E_corr_ (V)	Corrosion Rate (μm/Year)
Ti6Al4V	7.565 × 10^−8^	−0.608	23.8
T0	6.483 × 10^−7^	−0.264	204
T1	4.540 × 10^−7^	−0.318	143
T2	7.454 × 10^−8^	−0.319	23.4
T4	7.645 × 10^−8^	−0.320	24
T4-P	4.507 × 10^−7^	−0.465	142
T4-G	1.497 × 10^−7^	−0.646	47

**Table 4:** Fitted circuit values for untreated, mechanically ground and polished, and nitriding samples.

	R_s_	R_1_	CPE_1_	R_2_	CPE_2_	R_ct_	CPE_dl_
Ti6Al4V	91.3					1.67 × 10^6^	2.76 × 10^−5^
T0	105.6	76.2	2.43 × 10^−5^	446	1.13 × 10^−5^	3.98 × 10^5^	4.38 × 10^−5^
T1	101.3	57.3	3.11 × 10^−5^	635	1.17 × 10^−5^	1.07 × 10^6^	2.66 × 10^−5^
T2	104	320.8	4.13 × 10^−5^	12,417	5.58 × 10^−6^	8.61 × 10^6^	2.59 × 10^−5^
T4	94.5	6493.0	7.43 × 10^−5^	38,371	8.85 × 10^−6^	3.10 × 10^6^	7.11 × 10^−5^
T4-P	93.0	350.9	3.00 × 10^−5^			1.03 × 10^6^	1.28 × 10^−5^
T4-G	87.3	1805.0	3.32 × 10^−5^			1.65 × 10^6^	7.62 × 10^−5^

## Data Availability

Not applicable.
